# *Notes from the Field:* Congenital Varicella Syndrome Case — Illinois, 2021

**DOI:** 10.15585/mmwr.mm7110a5

**Published:** 2022-03-11

**Authors:** Jessica Leung, Heather D. Reid, Jodi Morgan, Heather Kadyk, Gayla Havener, Mona Marin

**Affiliations:** ^1^Division of Viral Diseases, National Center for Immunization and Respiratory Diseases, CDC; ^2^Illinois Department of Public Health; ^3^Sangamon County Health Department, Springfield, Illinois.

On April 8, 2021, a newborn was delivered at 24 weeks’ gestation with congenital varicella syndrome, after maternal varicella was diagnosed at 12 weeks’ gestation. At 22 weeks’ gestation, an ultrasound identified a multitude of fetal abnormalities (Box); congenital varicella syndrome was confirmed by a positive varicella-zoster virus (VZV) polymerase chain reaction test of the amniotic fluid. Because the prognosis of the fetus was poor, a decision was made to induce labor. At delivery, the newborn had a heart rate of 60 beats/minute, an Apgar score of 1, and weighed 526 g; the newborn died approximately 15 minutes after delivery. After birth, neither additional VZV testing nor an autopsy was performed.

BOX Timeline for congenital varicella syndrome case, by date and gestational age* — Illinois, 2021

**December 2, 2020 (gestational age: 5 weeks, 6 days)**

- Pregnant woman (patient) initiated routine prenatal care.
- Baseline maternal laboratory test result showed serology for VZV IgG antibodies was equivocal (value <135 [reference value for positive VZV >185]), rubella immune, and rapid plasma reagin nonreactive.
- Genetic screening results were negative for trisomy 13, 18, 21, and sex chromosome aneuploidy.

**December 3, 2020 and December 17, 2020 (gestational age: 6 weeks, 0 days and 8 weeks, 0 days)**

- Ultrasound was performed to estimate gestational age because of discrepancy with LMP.
- Estimated delivery date using LMP was June 30, 2021, and by ultrasound was July 29, 2021.
- There was no information collected on fetal characteristics from either ultrasound.

**January 19, 2021 (gestational age: 12 weeks, 5 days)**

- Patient reported onset of skin rash.
- There was no evidence of household contacts with rash illness or known source of exposure.

**January 21, 2021 (gestational age: 13 weeks, 0 days)**

- Patient visited her primary care provider and was clinically diagnosed with varicella. She had a generalized maculopapulovesicular rash with 250–499 lesions on arms, face, head, trunk, and legs; no complications were noted. It is unknown whether the patient visited an obstetrician at this time.
- Symptomatic treatment was provided; no antivirals were administered.
- Serology test result for VZV IgM was positive.

**February 9, 2021 (gestational age: 15 weeks, 5 days)**

- Patient visited obstetrician for routine appointment; varicella skin lesions had resolved.

**March 8, 2021 (gestational age: 19 weeks, 4 days)**

- Ultrasound showed incomplete visualization of fetal anatomy.

**March 25, 2021 (gestational age: 22 weeks, 0 days)**

- Patient consulted a maternal fetal medicine specialist because of her diagnosis of varicella during pregnancy.
- Ultrasound result showed fetal abnormalities, including abnormal profile (small chin and suspected orbit anomaly), absent cavum septum pellucidum, left orbit/lens abnormality, abnormal flexion of arms and legs with no movement, complex cardiac defect, and echogenic bowel; suboptimal fetus’s abdominal cord insertion and sex identification. The estimated fetal weight was low at 408 g (13th percentile).
- Amniocentesis result showed amniotic fluid specimen was positive for VZV by polymerase chain reaction. Test results were negative for cytomegalovirus, toxoplasmosis, and parvovirus.
- No genetic studies were performed at patient’s request because of amniocentesis confirming congenital varicella syndrome.

**April 8, 2021 (gestational age: 24 weeks, 0 days)**

- Labor was induced.
- Infant was born with a heart rate of 60 beats per minute, an Apgar score of 1, and weighing 526 g; newborn died approximately 15 minutes after delivery.
- Antibody screening showed newborn was negative for direct antiglobulin IgG.
- Placenta pathology results showed fetal membranes with mild chronic subchorionic inflammation, singleton placental disc (131g); premature/second trimester chorionic villi with scattered intervillous fibrin aggregates and associated mild chronic lymphocytic and mononucleate infiltrates, which focally extended to the adjacent villi.
- No additional laboratory testing nor autopsy was performed on the newborn after birth.

**Abbreviations:** LMP = last menstrual period; IgG = immunoglobin G; IgM = immunoglobin M; VZV = varicella-zoster virus.

* Gestational age estimated using an estimated delivery date of July 29, 2021, based on ultrasound.

The mother, aged 27 years, was born outside the United States and had no documented history of varicella disease or vaccination. She was healthy with no remarkable past medical history. She initiated routine prenatal care at 5 weeks and 6 days’ gestation; serum collected at that time was VZV immunoglobulin (Ig) G equivocal. At 12 weeks and 5 days’ gestation, she developed a maculopapulovesicular rash and received a diagnosis of varicella from her health care provider. Serologic testing for VZV IgM was positive. The source of exposure was unknown. The mother worked in a large retail store. Her older child, a boy aged 2 years, had received 1 dose of varicella vaccine in 2019 at age 1 year. He did not have a known rash near the time his mother developed varicella. Birth of this older child occurred in a different state, and records were not available for review. It is not known whether the mother was assessed for varicella immunity during her previous pregnancy. 

Before the introduction of routine childhood varicella vaccination in 1995, approximately 4 million cases, 10,500–13,500 hospitalizations, and 100–150 deaths from varicella occurred annually in the United States (*1*). The U.S. varicella vaccination program has reduced the incidence of varicella by >90%, as well as community transmission of VZV.* However, this case illustrates that severe consequences of VZV infection might occur and underscores the importance of vaccination. Congenital varicella syndrome can lead to severe birth defects, including hypoplasia of an extremity, microcephaly, skin and ocular abnormalities, intellectual disability, and low birth weight (*1*). This syndrome is estimated to occur among 0.4%–2.0% of newborns born to women who develop varicella during the first or second trimester of pregnancy (*1*). Because most women of childbearing age are immune to VZV, congenital varicella syndrome is rare. Before introduction of the varicella vaccine, approximately 44 cases of congenital varicella syndrome were estimated to have occurred annually in the United States (*1*). This is the third reported case of congenital varicella syndrome in the United States since the varicella vaccination program started in 1995 (*2*) (J Leung and M Marin, CDC, unpublished data, 2021); however, underreporting is possible because congenital varicella cases are not nationally notifiable. An Australian study documented reduction in the incidence of congenital varicella syndrome after implementation of universal varicella vaccination of children at age 18 months (*3*).

This case reaffirms current Advisory Committee on Immunization Practices recommendations for preventing varicella that all adults be assessed for varicella immunity, and that those who do not have evidence of immunity^†^ should receive 2 doses of varicella vaccine, with special emphasis for adult groups at high risk, including nonpregnant women of childbearing age (*1*). Among non–U.S.-born adults, birth before 1980 is not considered evidence of varicella immunity because of the higher likelihood of these adults to be susceptible to varicella, especially those from tropical climates (*1*,*4*). All pregnant women should have prenatal assessment for varicella evidence of immunity, and postpartum vaccination should be recommended for susceptible women. It is important to assess and assure documentation of evidence of immunity with each pregnancy, in advance of future pregnancies. If susceptible pregnant women are exposed to VZV, varicella-zoster immune globulin (VariZIG) is recommended to prevent severe maternal disease and should be administered within 10 days of exposure (*5*); whether this step modifies infection in the fetus is uncertain although some evidence suggests that it might be beneficial for the fetus. This intervention is effective only if an exposure is identified. 

This case of congenital varicella syndrome is a reminder that varicella during pregnancy can cause severe outcomes and underscores the importance of assessing varicella immunity in adults, vaccinating nonimmune persons, as well as prenatal assessment and postpartum vaccination of susceptible women against varicella.

## Footnotes

* https://www.cdc.gov/vaccines/pubs/surv-manual/chpt17-varicella.html (Accessed March 25, 2021).

† Evidence of immunity to varicella includes 1) documentation of age-appropriate varicella vaccination, 2) laboratory evidence of immunity or confirmation of disease, 3) diagnosis or verification of a history of varicella or herpes zoster by a health care provider, and 4) birth in the United States before 1980. The last criterion is based on serologic evidence of VZV infection documented in most U.S. adults born before 1980. Birth in the United States before 1980 is not adequate evidence of immunity for health care workers, pregnant women, or persons with weakened immune system; these persons need to meet one of the other criteria for evidence of immunity.
